# Vedolizumab for the Management of Refractory Behçet’s Disease: From a Case Report to New Pieces of Mosaic in a Complex Disease

**DOI:** 10.3389/fimmu.2021.769785

**Published:** 2021-10-25

**Authors:** Marta Arbrile, Massimo Radin, Daniela Rossi, Elisa Menegatti, Simone Baldovino, Savino Sciascia, Dario Roccatello

**Affiliations:** Center of Research of Immunopathology and Rare Diseases—Coordinating Center of Piemonte and Valle d’Aosta Network for Rare Diseases, Department of Clinical and Biological Sciences, Complex Structure with University Management (SCDU) Nephrology and Dialysis, S. Giovanni Bosco Hospital and University of Turin, Turin, Italy

**Keywords:** vedolizumab, Behçet disease, biological drug, intestinal Behçet, biological therapy

## Abstract

**Objectives:**

When treating Behçet’s disease (BD), anti-tumor necrosis factor (TNF)-α agents have become a second-line therapy when conventional immunosuppressive drugs have failed. However, in the case of failure of treatment with anti-TNFα drugs, further options are limited. Based on previous reports of the efficacy of vedolizumab (VDZ) in inflammatory bowel diseases, we decided to administer VDZ to treat a patient with intestinal BD.

**Methods:**

We present the case of a 49-year-old female patient with BD. Her clinical manifestations included erythema nodosum, oro-genital ulcers, positive Pathergy test, positive HLA-B51, and biopsy-proven intestinal BD. The patient was unsuccessfully treated with conventional immunosuppressive and several biological agents.

**Results:**

Treatment with VDZ was started intravenously at a dose of 300 mg at 0, 2, and 6 weeks and then every 4 weeks.

After the second dose of VDZ, the patient reported a marked improvement of intestinal BD and a concomitant amelioration of arthralgia, erythema nodosum lesions and aphthosis. Clinical remission was achieved at 6 months after starting VDZ.

**Conclusion:**

VDZ might represent a valid option to treat patients with BD who are non-responsive to standard treatments or anti-TNFα agents, particularly, those cases with intestinal involvement.

## Introduction

First identified in 1937, Behçet’s disease (BD) is a multisystemic inflammatory condition often described as a part of the vasculitic spectrum, characterized by recurrent oral and genital aphthosis, skin lesions, uveitis, and, less frequently, neurologic, articular, and gastrointestinal involvement (1). BD is mostly prevalent in countries along the ancient “Silk Road”, from the Mediterranean area to the far East, where it is associated with a significant prevalence of the major histocompatibility complex antigen HLA-B51 (2, 3). Before the availability of biological agents, options for the treatment of BD were limited to corticosteroids and conventional immunosuppressive drugs (1). The recent off-label use of biologic drugs such as infliximab and adalimumab, two anti-TNFα monoclonal antibodies, has improved the therapeutic armamentarium for refractory cases (4, 5). Nevertheless, not all patients fully respond to anti-TNFα agents, and it is quite common to experience a loss of efficacy over time in patients who initially had a beneficial effect (6).

Vedolizumab is a new biologic agent with a specific intestinal tropism, recently approved for the treatment of inflammatory bowel diseases (IBDs) (7, 8). VDZ binds to the α4β7 integrin, a glycoprotein expressed on the cell surface of circulating B and T lymphocytes, and blocks the interaction between the integrin and the mucosal addressin cell adhesion molecule 1 on the endothelium of intestinal blood vessels (9). The rationale for the use of VDZ in BD patients relies on the similar gastro-intestinal involvement of the two conditions. In fact, a growing body of evidence is suggesting that IBDs and BD may be closely related and part of a common disease spectrum rather than distinct disease entities (10).

Herein we describe the case of a patient with BD with gastro-intestinal involvement refractory and/or intolerant to several previous therapeutic approaches (including both conventional and biological disease modifying anti-rheumatic drugs, DMARDs) but who was successfully treated with vedolizumab (VDZ) and represents, to our knowledge, the first report about the use of VDZ in BD.

## Case Report

We present the case of a 49-year-old female patient. Her clinical history was unremarkable, aside from cutaneous psoriasis until the age of 35 when, during the post-partum period of her second pregnancy, she presented with a new onset of fever, diarrhea, and ankle arthritis. The concomitant presence of erythema nodosum and oro-genital ulcers along with the negativity for antinuclear antibodies, anti-double-stranded DNA antibodies, and normal levels of C3 and C4 supported a BD diagnosis. Colchicine and low doses of corticosteroids were started, with improvement on the aphthosis and the erythema nodosum, but with no changes in the persistence of abdominalgia and diarrhea. After a careful investigation to rule out any concomitant infectious disease, treatment with cyclosporine was started, which initially only elicited limited benefit and was subsequently suspended due to the onset of dizziness and worsening of intestinal symptoms. The ileo-colonoscopy was consistent with ileocolitis with two sigmoid colon irregularities of aphtoid aspect adjacent to macroscopically normal-appearing mucosa. The colon biopsy results reported nonspecific chronic inflammation with follicular hyperplasia of the lymphoreticular tissue with no presence of granulomas, confirming the histological diagnosis of BD.

The list of main investigations undergone by the patient is presented in **Table 1**. The patient attended other centers, from 2011 to 2017, where she was treated with different cDMARDs and bDMARDs as follows: sulfasalazine, which was suspended for increased liver enzymes, while azathioprine, adalimumab, infliximab, golimumab, and certolizumab were all discontinued due to lack of response. Secukinumab, introduced in 2018, was stopped regardless of the beneficial effects on BD symptoms due to the development of an anxious–depressive syndrome with suicidal thoughts, which spontaneously subsided when secukinumab was suspended. In 2019, a further attempt with golimumab was performed, with no clinical benefit and no control on the persistence of erythema nodosum, aphthosis, and diarrhea.

**Table 1:** Previous investigations undergone by the patient.

Blood count	WBC 10,000 (cells/µl); neutrophils 7,210; lymphocytes 2,470; RBC 5,700; Hb 11.3 g/dl; MCV 62 fL; PLTs 248,000
Blood tests	Crea 0.74, AST 21, ALT 29, GGT 19, total protein 73, C3 1.65 g/l, C4 0.26 g/l, ESR 25, CRP 26, ANA pos 1:640, ENA neg, anti-dsDNA neg, antiphospholipid antibodies neg, HBV-DNA: neg; HCV-RNA: neg; Normal complete urine test, Uroculture: neg.
Shoulder MRI	Signs of acromioclavicular fibroarthrosis, compression of the myo-tendon tract of the supraspinatus in which the tendon shows a inhomogeneous character in the pre-insertion anterior area due to tendinosis; signal alteration of the medullary bone component of the humeral shaft
Cerebral MRI	Unremarkable
Pelvis MRI	Initial sacrum—ileitis of the right sacro-iliac joint
Abdominal echography	Steatotic liver disease, free of focal lesions. Outcomes of cholecystectomy. Mild hypersplenomegaly (diameter 12.5 cm). Anechoic oval formation at uterine level with a diameter of 18 mm, compatible with a follicle
Abdominal TC	Thickening of the wall of the small intestine. At the level of an intestinal loop in the left iliac area between the bladder and the acetabular region, the wall appears markedly thickened, inhomogeneous, and with discrete effusion.
Colonoscopy/Colon biopsy	Mucosal irregularities of aphtoid aspect at the sigmoid colon level. Mucosal flaps of the large intestine with mild atrophy, chronic interstitial inflammation, edema, and hyperplasia of the muscularis mucosae.

**Figure 1:**
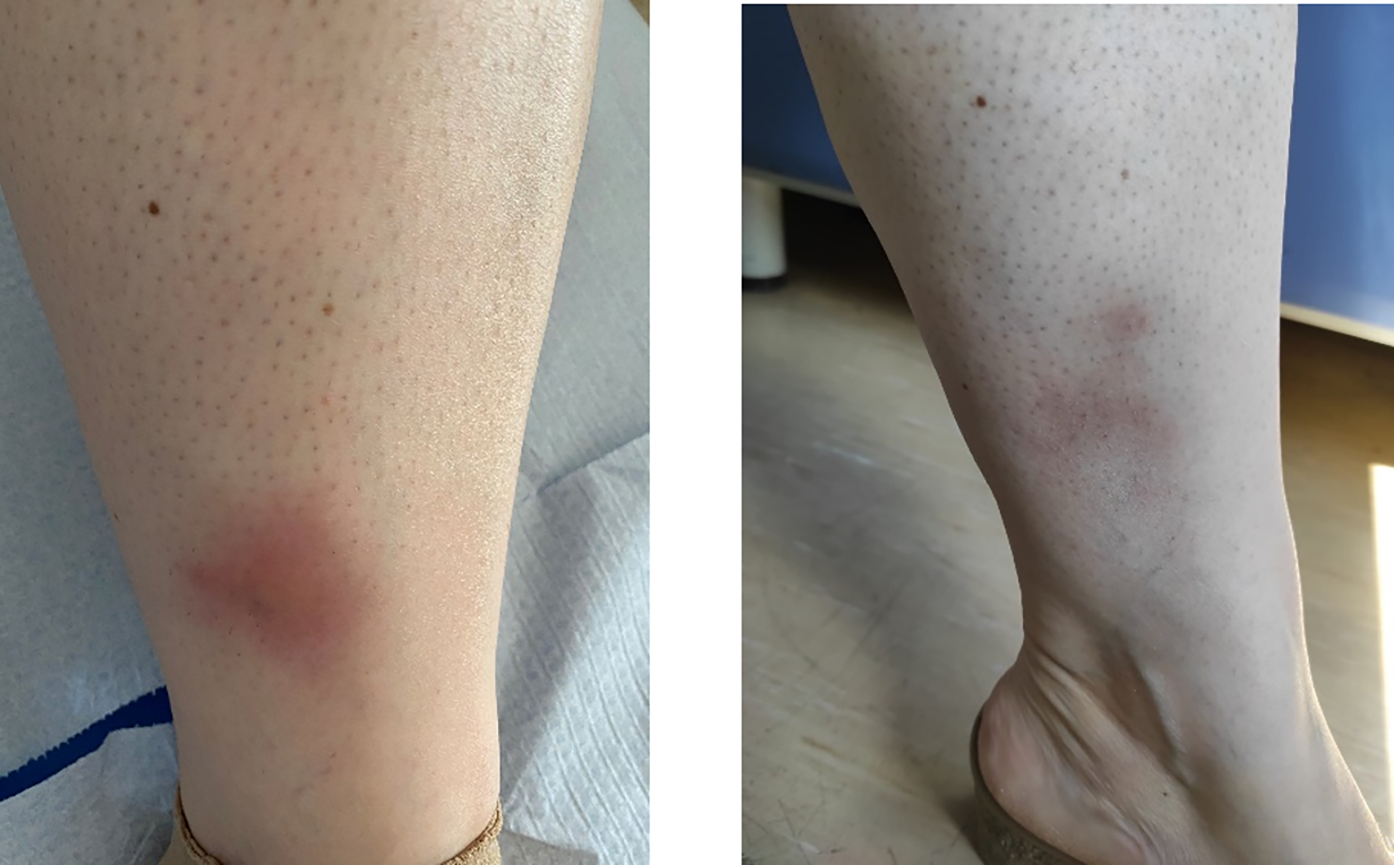
Active tibial erythema nodosum at the first presentation of the patient, before starting with vedolizumab (left panel) and after 3 months (right panel).

In November 2020, the woman was referred to our center (CMID, S. Giovanni Bosco Hub, Turin, Italy) for further evaluation. She presented with arthralgia (mainly affecting the wrists, metacarpophalangeal joints, and shoulders) and recurrent ulcerations and folliculitis on her back, forehead, and malar area. During the first evaluation, her main complaints were abdominal pain and persistent diarrhea which constantly impact her quality of life and were only partially responsive to symptomatic treatment. The physical examination showed two areas of erythema nodosum on the lower limbs (**Figure 1**). The Pathergy test and HLA-B51 were positive. Her laboratory profile was consistent with active BD, characterized by markedly increased erythrocyte sedimentation rate and C-reactive protein (26 mg/dl). After a multidisciplinary evaluation, taking into account the scarce efficacy of both cDMARDs and anti-TNFα strategies and the persistence of gastrointestinal symptoms, we decided to start treatment with VDZ, which was administered intravenously in monotherapy at a dose of 300 mg at 0, 2, and 6 weeks and then every 4 weeks.

After the second dose of VDZ, the patient reported a marked improvement when considering gastrointestinal symptoms and arthralgia, with a decrease in the Behçet’s Disease Current Activity Form 2006 (11) score from 6 (at the VDZ starting time) to 0 (after 6 months of follow-up). A significant reduction of the multiple erythema nodosum lesions affecting both legs was progressively observed, with a complete resolution after the third dose and parallel amelioration of the associated pain.

At 6 months of follow-up, no side effects were observed, and the patient did not experience further episodes of aphthosis.

## Discussion

In this study, we reported the case of a woman with BD complicated by biopsy-proven gastrointestinal involvement, who was unresponsive to conventional treatment and various anti-TNFα agents, and who experienced a marked resolution of the gastrointestinal symptoms when treated with VDZ.

The patient was diagnosed with BD in 2007 because of recurrent oral and genital aphthosis, erythema nodosum, arthritis, and intestinal involvement. Since BD diagnosis is not characterized by specific laboratory, radiologic, or histologic findings, the diagnosis of this condition relies mainly on clinical evaluation (1). The most recent classification criteria are the International Criteria for Behçet Disease, published in 2006 and revised in 2014, which require a score of 4 from the following list: oral aphthosis, genital aphthosis, ocular manifestations, skin involvement, vascular manifestations, neurologic manifestation, and positive Pathergy test (12). Gastrointestinal involvement in BD remains infrequent. Interestingly, geographical differences among BD patients have been reported, with colon involvement being more frequent in patients from Europe and North America, while ileocecal involvement appears to be more common in Japan (13).

**Figure 2:**
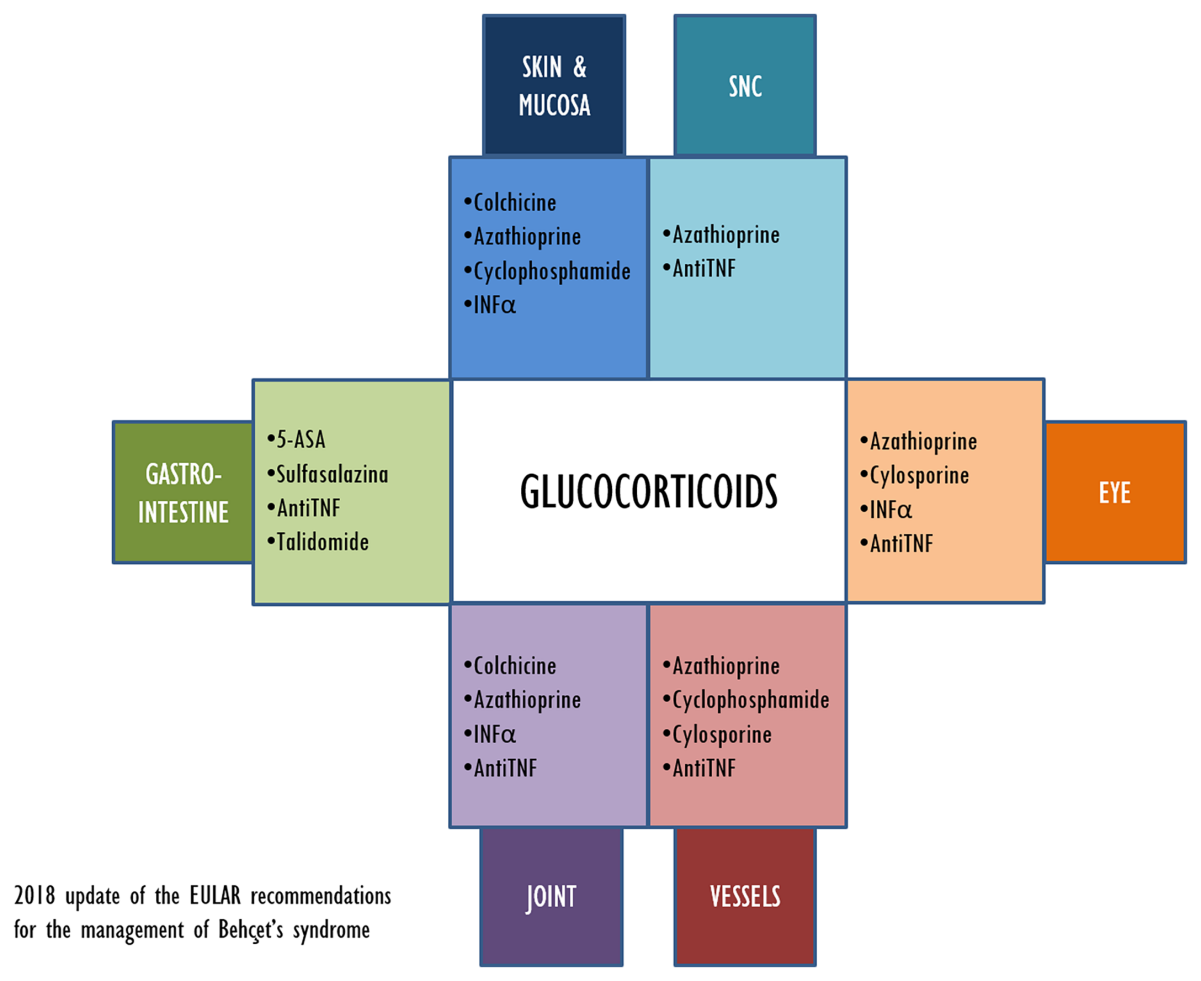
2018 update of the EULAR recommendations for the management of Beçhet’s syndrome.

The aim of the treatment in BD is maintaining remission and improving the quality of life of the patient, but new therapeutic approaches are still needed. **Figure 2** illustrates the current therapeutic options for BD treatment and their main application related to clinical symptoms.

For many years, the only bDMARDs available for the management of BD were anti-TNFα agents. More recently, anti-cytokine-related (*e*.*g*., IL-6) strategies have been suggested as therapeutic options for BD, with a good safety and efficacy profile (14, 15). Recently approved for IBDs, VDZ, targeting a completely novel mechanism of action, could represent a new tool in the therapeutic armamentarium of BD specialists. VDZ can be considered as an “integrin antagonist” due to its ability of binding to the α4β7 integrin (a molecule identifiable on T-lymphocytes and mononuclear cells). This gastrointestinal-specific interaction reduces the side effects associated with systemic immunosuppression. The severe intestinal involvement of the patient discussed in our study was the main reason for switching to VDZ from anti-TNFα agents. Despite the specific gastrointestinal effect, in several studies VDZ has proved to be effective also when managing the extraintestinal manifestations of IBDs (*i*.*e*., skin involvement, uveitis, and arthritis), even if the mechanism remains unknown to this day (16, 17). These observations are in line with our case, as during follow-up, after 6 months of treatment with VDZ, our patient remained asymptomatic and without any sign of active inflammation. Of note is that no side effects were reported.

Meant to prevent the migration of inflammatory mononuclear cells into the inflamed mucosa in patients with IBD, VDZ could also mitigate the inflammatory process in BD (3), limiting the interaction of lymphocyte integrins (as expressed by CD4+ and CD8+ naive T cells and CD4+ and CD8+ memory T cells) and their endothelial ligands (18).

## Conclusion

In conclusion, this case shows the efficacy of VDZ in inducing remission at 6 months of follow-up in a patient not responsive to anti-TNFα agents. When treating BD, the anti-TNFα agents have become a standard when conventional immunosuppressive drugs have failed. However, in case of anti-TNFα agents failure, the options are limited. A switch to another anti-TNFα drug is a current possibility, but in our case it was not effective. Based on previous reports of its efficacy in IBDs, we decided to administer VDZ to treat the intestinal involvement of our patient. This led not only to a satisfactory gastrointestinal response but also to the concomitant disappearing of ulcerations, arthralgia, and a reversion of the skin lesions.

Some limitations should be acknowledged, mainly the short follow-up which is currently up to 6 months. Further larger cohort studies with a longer follow-up are needed to provide more evidence on these preliminary findings, especially in regard to the dosage of VDZ, the duration of treatment, the compatibility with other immunosuppressive agents, and the efficacy on patients without intestinal involvement. However, VDZ might represent a valid option for the treatment of patients with BD who are not responsive to standard treatment or anti-TNF agents.

## Data Availability Statement

The raw data supporting the conclusions of this article will be made available by the authors, without undue reservation.

## Ethics Statement

Ethical approval was not provided for this study on human participants because the study is a case report. Written consensus was retrieved by the patient and the research was run in accordance with the declaration of Helsinki. The patients/participants provided their written informed consent to participate in this study.

## Author Contributions

MA participated in data collection, interpretation of results, and drafting of the manuscript. MR, DRos, EM, SB, SS, and DRoc participated in data collection, interpretation of results, manuscript preparation, and final review. All authors contributed to the article and approved the submitted version.

## Conflict of Interest

The authors declare that the research was conducted in the absence of any commercial or financial relationships that could be construed as a potential conflict of interest.

## Publisher’s Note

All claims expressed in this article are solely those of the authors and do not necessarily represent those of their affiliated organizations, or those of the publisher, the editors and the reviewers. Any product that may be evaluated in this article, or claim that may be made by its manufacturer, is not guaranteed or endorsed by the publisher.
